# Low alanine aminotransferase levels are independently associated with mortality risk in patients with atrial fibrillation

**DOI:** 10.1038/s41598-022-16435-5

**Published:** 2022-07-16

**Authors:** Yuki Saito, Yasuo Okumura, Koichi Nagashima, Daisuke Fukamachi, Katsuaki Yokoyama, Naoya Matsumoto, Eizo Tachibana, Keiichiro Kuronuma, Koji Oiwa, Michiaki Matsumoto, Toshihiko Nishida, Toshiaki Kojima, Shoji Hanada, Kazumiki Nomoto, Kazumasa Sonoda, Ken Arima, Fumiyuki Takahashi, Tomobumi Kotani, Kimie Ohkubo, Seiji Fukushima, Satoru Itou, Kunio Kondo, Hideyuki Ando, Yasumi Ohno, Motoyuki Onikura, Atsushi Hirayama

**Affiliations:** 1 Division of Cardiology, Department of Medicine, Nihon University School of Medicine, 30-1 Oyaguchi-kamicho, Itabashi-ku, Tokyo, 173-8610 Japan; 2 Department of Cardiology, Nihon University Hospital, Tokyo, Japan; 3 Kawaguchi Municipal Medical Center, Saitama, Japan; 4 Yokohama Chuo Hospital, Kanagawa, Japan; 5 Sekishindo Hospital, Saitama, Japan; 6 Asakadai Central General Hospital, Saitama, Japan; 7 Tokyo Rinkai Hospital, Tokyo, Japan; 8 Kasukabe Municipal Hospital, Saitama, Japan; 9 Yasuda Hospital, Tokyo, Japan; 10 Makita General Hospital, Tokyo, Japan; 11 Itabashi Medical Association Hospital, Tokyo, Japan; 12 Ukima Central Hospital, Tokyo, Japan; 13 Itou Cardiovascular Clinic, Saitama, Japan; 14 Kondo Clinic, Tokyo, Japan; 15 Keiai Clinic, Tokyo, Japan; 16 Ohno Medical Clinic, Tokyo, Japan; 17 Onikura Clinic, Chiba, Japan

**Keywords:** Cardiology, Medical research

## Abstract

Extremely low alanine aminotransferase (ALT) may reflect aging, frailty, sarcopenia, and malnutrition in several cardiovascular diseases, but the association between low ALT and patient characteristics, cardiovascular and all-cause mortality is not well investigated in the population with atrial fibrillation. We conducted a post hoc analysis of a prospective, observational multicenter study. Patients with nonvalvular AF in the SAKURA AF Registry (n = 3156) were classified into 3 tertiles according to baseline ALT: first (ALT ≤ 15 U/L, n = 1098), second (15 < ALT < 23 U/L, n = 1055), and third (ALT ≥ 23 U/L, n = 1003). The first tertile had an older age; lower body mass index (BMI); higher prevalence of heart failure; and lower hemoglobin, total cholesterol, and triglycerides (all *P* < 0.05). During median 39.2 months follow-up, the first tertile had significantly higher incidences of cardiovascular and all-cause mortality (log-rank *P* < 0.001). Lower ALT was significantly associated with the incidence of cardiovascular and all-cause mortality, even after adjusting for clinically relevant factors (*P* < 0.05). Low ALT may reflect aging, sarcopenia, and malnutrition and be independently associated with a high risk of all-cause mortality in patients with AF.

## Introduction

Atrial fibrillation (AF) is the most common type of arrhythmia in the older population, and the prevalence of AF is increasing, mainly because of the aging society^1^. Aged people frequently have comorbidities such as hypertension and diabetes mellitus, and other cardiovascular diseases, therefore AF is not a simple one of the arrhythmias, and its prognosis is significantly affected by the AF comorbidities^2^.

The presence of AF is strongly associated with a high risk of ischemic stroke, congestive heart failure, and mortality^3,4^. Among these adverse events, AF is strongly associated with mortality^5^. Several recent studies have shown that patients with AF had a 3 to 5-hold higher risk of cardiovascular and all-cause mortality than the general population^6,7^. As the burden of AF on healthcare systems continues to increase, risk stratification by identifying high-risk patients is essential for long-term management strategies to reduce the burden.

Patients of advanced age with AF usually have complex comorbidities, frailty, and malnutrition status. Recently, the relationships between physical activity, nutritional status, and clinical outcomes of AF have attracted the interest of researchers^8,9^. Patients with malnutrition are more likely to develop AF^10^. In addition, several recent studies showed that malnutrition was significantly associated with cardiovascular events, recurrence of AF after catheter ablation, and all-cause mortality^11,12^.

Gluconeogenesis, the essential metabolic step to produce glucose and energy that occurs mainly in the liver and skeletal muscles, is facilitated by the enzyme alanine aminotransferase (ALT)^13^. ALT is predominantly found in the liver and catalyzes the conversion of alanine into α-ketoacids. Serum ALT is commonly measured in clinical practice, and a higher level is found in liver and skeletal muscle diseases, presumably as a result of the release of the enzyme into the blood by damaged cells^14,15^. Non-alcoholic fatty liver disease (NAFLD), which was recently revealed to be a risk factor for the development of AF, is a manifestation of metabolic syndrome and could cause mild elevation of ALT^16^. In contrast, recent studies indicated that an extremely low ALT level might reflect the process of aging, frailty, sarcopenia, and malnutrition in older people, indicating another clinically relevant reason to measure this enzyme^14^. In addition, other studies reported that low ALT is associated with aging, frailty, malnutrition, and higher mortality in the older population and with several cardiovascular diseases^13,17^. Nevertheless, the relation between low ALT and patient characteristics and risk of cardiovascular and all-cause mortality is not well investigated in patients with AF. Therefore, the present study aimed to investigate the association of low ALT with the severity of malnutrition and sarcopenia and with mortality risk in patients with AF.

## Methods

### Study population

Our study was a post hoc analysis of a prospective, observational multicenter study on the SAKURA AF Registry (UMIN000014420)^18,19^. The SAKURA AF Registry was set up to support multicenter, prospective observational research by tracking clinical events in patients with AF for at least 2 years and up to 4 years after their enrollment in the registry. The study design, data collection process, and baseline characteristics of the study population have been reported elsewhere^18,19^. A total of 3267 patients with nonvalvular AF were enrolled into the registry by 63 institutions (2 cardiovascular centers, 20 affiliated hospitals or community hospitals, and 41 private clinics) in the Tokyo area from September 2013 until December 2015. Analysis of the registry data was approved by our Institutional Review Board (IRB) and by the IRBs of the individual hospitals. All enrollees provided written informed consent for inclusion in the registry. Baseline serum ALT values were missing for 111 patients in the registry, so these patients were excluded from the present study, and the data of the remaining 3156 patients were analyzed.

### Data collection and outcomes

Patient information was collected by a web-based registration system, which was accessed through the SAKURA AF Registry website. Relevant clinical data, such as comorbidities, medication use, and laboratory test results, and biannual follow-up information, such as laboratory data, were obtained. Serum ALT levels at enrollment in the registry (baseline) were used to classify patients into 3 groups according to the tertile of ALT values.

The type of AF was categorized as paroxysmal, persistent, or long-standing persistent, whereby paroxysmal AF was defined as AF lasting for up to 7 days. A history of heart failure was defined as a diagnosis of heart failure before enrollment of the patient in the registry. Creatinine clearance (CrCl) was estimated by the Cockcroft–Gault formula^20^. To assess nutritional status, we calculated the Triglycerides × Total Cholesterol × Body Weight Index (TCB index), a newly developed nutritional index that has been validated in patients with coronary artery disease or acute decompensated heart failure and in critical patients with cardiovascular disease^21–23^. The TCB index is calculated from triglyceride and total cholesterol levels and body weight by the following formula: TCB index = triglycerides (mg/dL) × total cholesterol (mg/dL) × body weight (kg)/1000^21^.

The primary endpoints of this study were cardiovascular and all-cause mortality. We also recorded the incidences of strokes and major bleeding. Stroke outcomes included ischemic stroke, hemorrhagic stroke, and transient ischemic attack (TIA). Major bleeding was defined as hemoglobin reductions of at least 2 g/dL, the transfusion of at least 2 units of blood, or symptomatic bleeding in a critical area of an organ.

### Statistical analysis

Categorical variables are shown as numbers (percentage), and continuous variables are shown as mean ± standard deviation (SD). The statistical differences in categorical variables between the 3 groups were tested by Chi-square test with Bonferroni correction, and those in continuous variables were calculated by one-way analysis of variance, followed by the post hoc Tukey–Kramer test or the Kruskal–Wallis test and the Steel–Dwass test. The occurrence of the endpoint is presented as Kaplan–Meier cumulative survival curves and was compared by the log-rank test. The Cox-proportional hazard model was used to analyze the relationship of ALT with event incidences, and hazard ratios with 95% confidence intervals were calculated. For the multivariable analysis, we used the clinically relevant factors as explanatory variables. Furthermore, we evaluated the non-linear relationship between ALT levels and the incidence of all-cause mortality using restricted cubic spline curves with cox proportional hazard model. The C statistic, net reclassification improvement, and integrated discrimination improvement were calculated to evaluate whether the accuracy of predicting all-cause mortality incidences would improve after adding ALT (as a continuous variable) to a baseline model with the CHA_2_DS_2_-VASc score, which was recently used as a predictor of all-cause mortality in AF patients^24^. All statistical analyses were performed with JMP 13.0 (SAS Institute, Cary, NC, USA) and R Statistics version 4.0.2 (R Foundation for Statistical Computing, Vienna, Austria). For all analyses, *P* < 0.05 was considered statistically significant.

### Ethics approval and consent to participate

This study was conducted in accordance with the principles of the Declaration of Helsinki, and with the approval of IRB of the Nihon University Itabashi Hospital and the IRBs of the individual hospitals. The study design was registered with UMIN (UMIN000014420). All participants gave their written informed consent prior to participation.

## Results

### Baseline characteristics

The median (interquartile range (IQR)) ALT value in the 3156 patients was 18 (14, 25) U/L. The patients were categorized into 3 tertiles according to the baseline ALT values, and the cut-off values of ALT between tertiles were determined to be 15 U/L and 22 U/L: first tertile (ALT ≤ 15 U/L, n = 1098), second tertile (15 < ALT < 23 U/L, n = 1055), and third tertile (ALT ≥ 23 U/L, n = 1003). The baseline characteristics of the study population are presented in Table 1. Compared with the other 2 tertiles, the first tertile had an older age; lower BMI; higher prevalence of BMI < 18.5 kg/m^2^, women, and heart failure; and higher CHADS_2_ and CHA_2_DS_2_-VASc scores (all *P* < 0.05). The first tertile also had lower hemoglobin, total cholesterol, triglycerides, TCB index, aspartate aminotransferase (AST), and CrCl and higher blood urea nitrogen (all *P* < 0.05). Administration of antiplatelets, warfarin, and direct-acting oral anticoagulants was similar in the 3 tertiles.

**Table 1:** Baseline patient characteristics according to baseline alanine aminotransferase level.

Item	Patients in first tertile (ALT ≤ 15 U/L), n = 1098	Patients in second tertile (15 < ALT < 23 U/L), n = 1055	Patients in third tertile (ALT ≥ 23 U/L), n = 1003	*P* value
**Baseline clinical data**				
Age, y	74.7 ± 8.6	72.0 ± 9.0*	68.8 ± 9.4*^†^	< 0.001
Male, n (%)	747 (68.0)	777 (73.6)*	802 (79.9)*^†^	< 0.001
Body mass index, kg/m^2^	23.3 ± 3.5	23.9 ± 3.5*	24.9 ± 4.0*^†^	< 0.001
Body mass index < 18.5 kg/m^2^, n (%)	71 (6.5)	45 (4.3)*	32 (3.2)*	< 0.001
AF type				0.12
Paroxysmal AF	412 (37.5)	389 (36.8)	360 (35.8)	–
Persistent AF	238 (21.6)	221 (20.9)	247 (24.6)	–
Long-standing persistent AF	433 (39.4)	440 (41.7)	388(38.6)	–
**Comorbidities**				
Type 2 diabetes, n (%)	233 (21.2)	227 (21.5)	258 (25.7)*	0.025
Hypertension, n (%)	792 (72.1)	746 (70.7)	717 (71.4)	0.76
History of heart failure, n (%)	282 (25.6)	212 (20.0)*	205 (20.4)*	0.002
Vascular disease, n (%)	166 (15.1)	119 (11.2)*	103 (10.2)*	0.001
History of stroke/TIA, n (%)	126 (11.4)	124 (11.7)	101 (10.0)	0.43
CHADS_2_ score	1.9 ± 1.1	1.7 ± 1.1*	1.6 ± 1.1*	< 0.001
CHA_2_DS_2_-VASc score	3.3 ± 1.4	2.9 ± 1.4*	2.6 ± 1.4*^†^	< 0.001
**Medications**				
Antiplatelet use	194 (17.6)	161 (15.2)	145 (14.4)	0.10
DOAC use	554 (50.4)	567 (53.7)	527 (52.5)	0.30
Warfarin use	544 (49.5)	488 (46.2)	476 (47.4)	0.30
**Laboratory data**				
Hemoglobin, g/dL	13.0 ± 1.5	13.8 ± 1.5*	14.3 ± 1.6*^†^	< 0.001
Platelet count, × 10^3^/μL	201 ± 56	200 ± 53	197 ± 52	0.24
Total cholesterol, mg/dL	182 ± 32	186 ± 31*	186 ± 32	0.029
Triglycerides, mg/dL	117 ± 67	136 ± 88*	157 ± 123*^†^	< 0.001
TCB index	1383 ± 1054	1689 ± 1278*	2141 ± 2625*^†^	< 0.001
AST, U/L	20 ± 4	24 ± 5*	35 ± 18*^†^	< 0.001
BUN, mg/dL	18 ± 8	17 ± 6	17 ± 7*^†^	< 0.001
CrCl, mL/min	59 ± 22	67 ± 23*	77 ± 29*^†^	< 0.001

Values are shown as mean ± SD or number (%), unless otherwise indicated.

For multiple comparisons, analysis of variance was used for symmetric continuous variables, the Kruskal–Wallis test was used for asymmetric continuous variables, and the Chi-squared test was used for categorical variables. All pairwise comparisons were performed with the Tukey–Kramer test for symmetric continuous variables, the Steel–Dwass test for asymmetric continuous variables, and the Chi-squared test with Bonferroni correction for categorical variables.

*P < 0.05 vs first tertile, ^†^*P* < 0.05 vs second tertile.

*AF* atrial fibrillation, *ALT* alanine aminotransferase, *AST* aspartate aminotransferase, *BUN* blood urea nitrogen, *CHADS*_*2*_ congestive heart failure, hypertension, age ≥ 75 years, diabetes, and stroke, *CHA*_*2*_*DS*_*2*_*-VASc* congestive heart failure, hypertension, age ≥ 75 years, diabetes, stroke, vascular disease, age 65–74 years, and male, *CrCl* creatinine clearance, *DOAC* direct oral anticoagulant, *TIA* transient ischemic attack.

### Clinical outcomes

**Figure 1:**
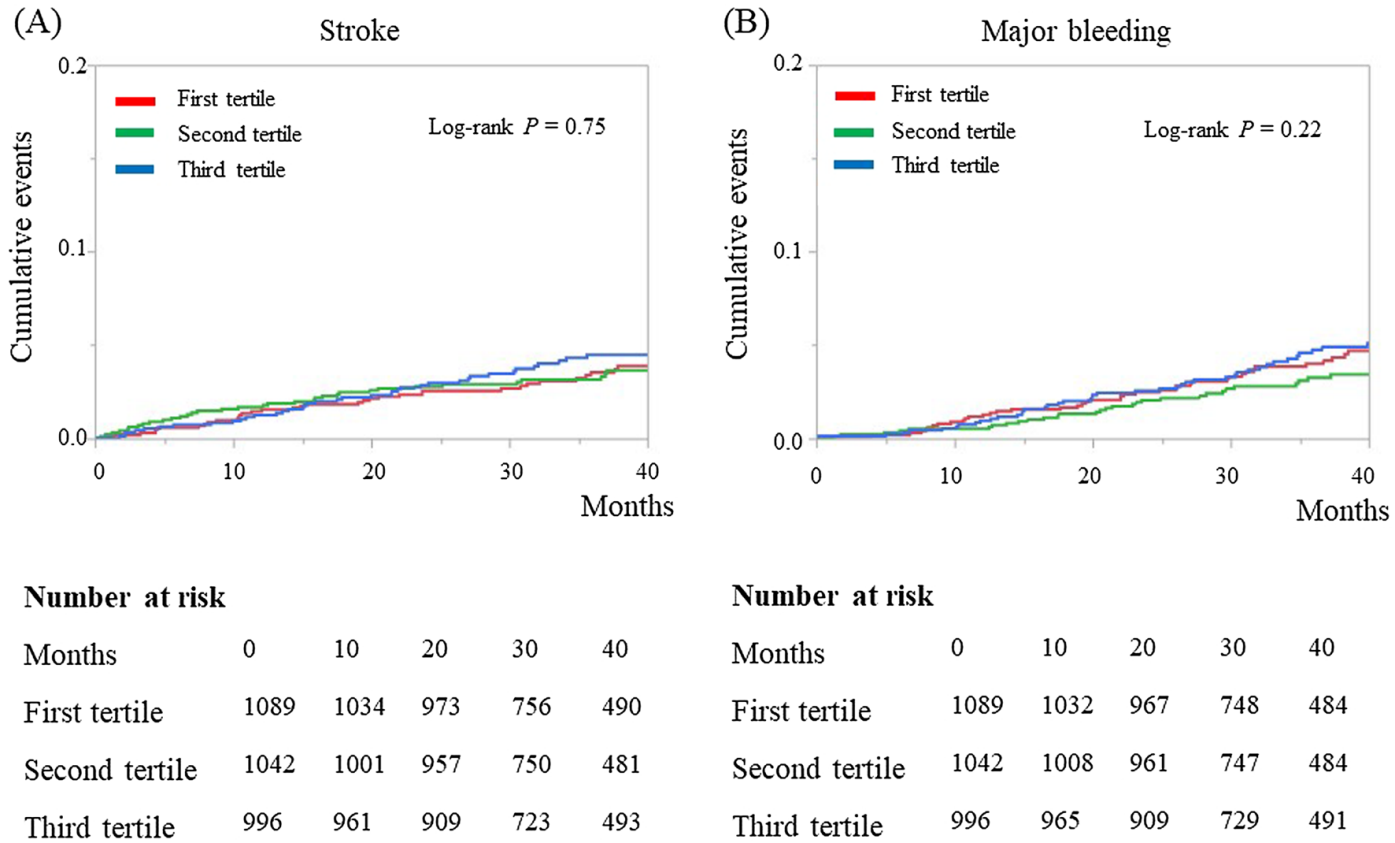
Kaplan–Meier curves for the incidence of stroke (**A**) and major bleeding (**B**) during the follow-up period according to the tertiles of baseline alanine aminotransferase values in patients with nonvalvular atrial fibrillation (n = 3156) in the SAKURA AF Registry.

**Figure 2:**
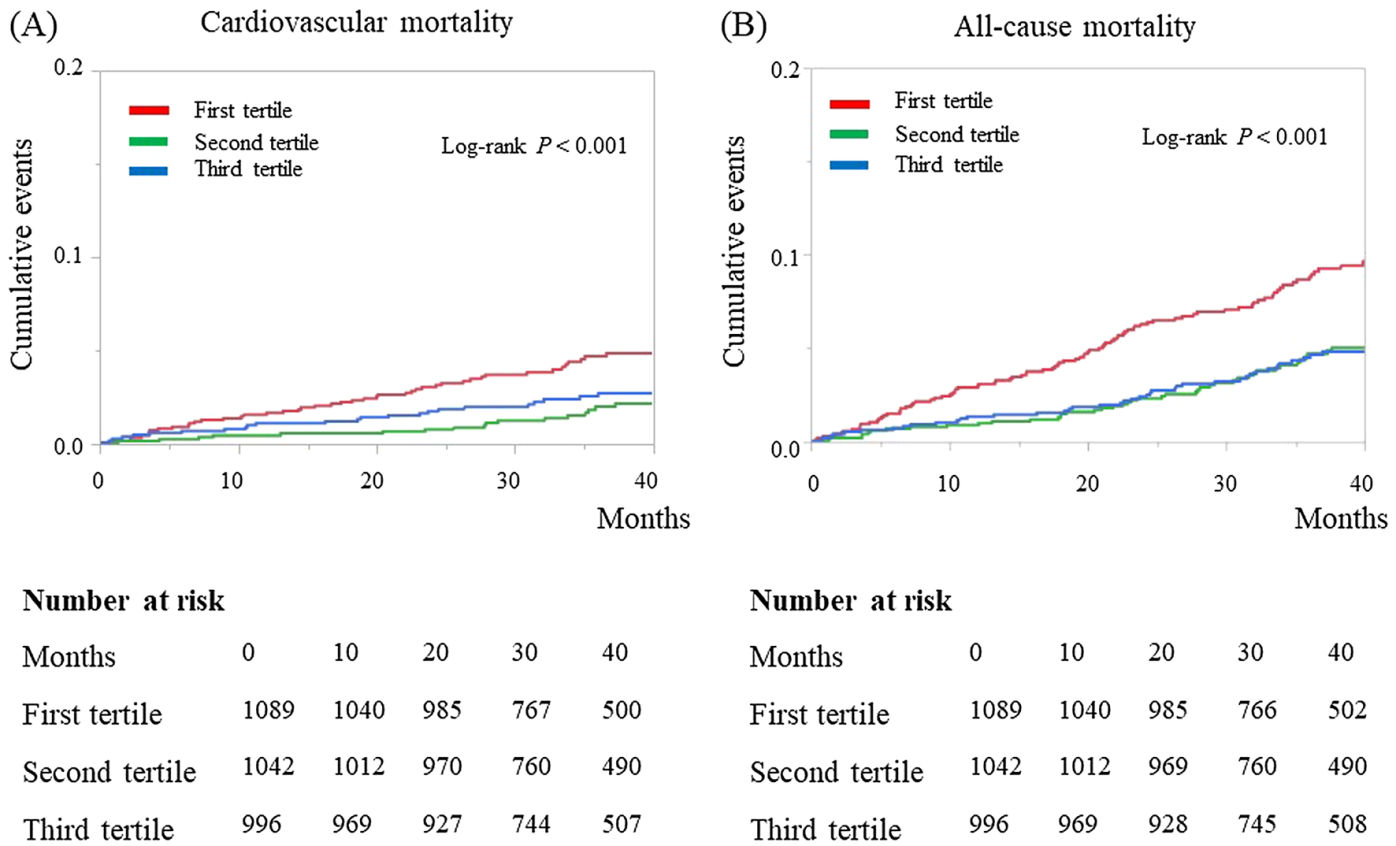
Kaplan–Meier curves for the incidence of cardiovascular mortality (**A**) and all-cause mortality (**B**) during the follow-up period according to the tertiles of baseline alanine aminotransferase values in patients with nonvalvular atrial fibrillation (n = 3156) in the SAKURA AF Registry.

During the median follow-up period of 39.2 months (IQR, 28.4, 43.6 months), 119 (3.8%) patients had a stroke event, 122 (3.9%) had major bleeding, 91 (2.9%) had cardiovascular death, and 196 (6.2%) had all-cause death. Kaplan–Meier curves for stroke and major bleeding are presented in Fig. 1, and Kaplan–Meier curves for cardiovascular and all-cause mortality are presented in Fig. 2. The incidences of stroke and major bleeding were not significantly different between the 3 tertiles (stroke, log-rank *P* = 0.75; major bleeding, log-rank *P* = 0.22). However, the incidence of cardiovascular mortality and all-cause mortality were significantly higher in the first tertile than in the second and third tertiles (log-rank P < 0.001).

On univariate Cox proportional regression analysis, neither lower ALT as a continuous variable nor ALT as a categorical variable (first vs second and third tertile) was significantly associated with the incidences of stroke and major bleeding (Table 2). Both lower ALT as a continuous variable and ALT as a categorical variable were significantly associated with the incidence of cardiovascular mortality; after adjusting for the other clinically relevant factors, ALT as a categorical variable remained associated with the incidence of cardiovascular mortality. In addition, both lower ALT as a continuous variable and ALT as a categorical variable were significantly associated with the incidence of all-cause mortality, even after adjusting for the other clinically relevant factors (all *P* < 0.05, Table 2).

**Table 2:** Adverse clinical outcomes and results of the Cox proportional regression model according to tertile of baseline alanine aminotransferase level.

Variable	Univariate analysis		Multivariate analysis**	
	HR (95% CI)	*P* value	HR (95% CI)	*P* value
**Stroke**				
ALT as a categorical variable (first vs second and third tertile*)	0.96 (0.65–1.40)	0.85	0.83 (0.56–1.23)	0.36
ALT as a continuous variable (per 1 U/L increase)	1.00 (0.98–1.01)	0.94	1.00 (0.99–1.01)	0.35
**Major bleeding**				
ALT as a categorical variable (first vs second and third tertile*)	1.18 (0.81–1.69)	0.37	1.08 (0.73–1.560)	0.68
ALT as a continuous variable (per 1 U/L increase)	1.00 (0.99–1.01)	0.60	1.00 (0.99–1.01)	0.28
**Cardiovascular mortality**				
ALT as a categorical variable (first vs second and third tertile*)	2.26 (1.49–3.41)	< 0.001	1.69 (1.10–2.59)	0.016
ALT as a continuous variable (per 1 U/L increase)	0.97 (0.94–0.99)	0.006	0.98 (0.96–1.01)	0.23
**All-cause mortality**				
ALT as a categorical variable (first vs second and third tertile*)	1.96 (1.48–2.60)	< 0.001	1.53 (1.14–2.04)	0.003
ALT as a continuous variable (per 1 U/L increase)	0.96 (0.94–0.98)	< 0.001	0.98 (0.96–0.99)	0.015

*First tertile, baseline ALT ≤ 15 U/L, n = 1098; second tertile, 15 < ALT < 23 U/L, n = 1055; and third tertile, ALT ≥ 23 U/L, n = 1003.

**Adjusted for age (≥ 75 years), sex, lower body weight (< 50 kg), AF type (persistent or long-standing persistent AF vs paroxysmal AF), hypertension, type 2 diabetes, heart failure, vascular disease, lower creatinine clearance (≤ 50 mL/min), history of stroke/TIA.

*ALT* alanine aminotransferase, *HR* hazard ratio, *CI* confidence interval.

**Figure 3:**
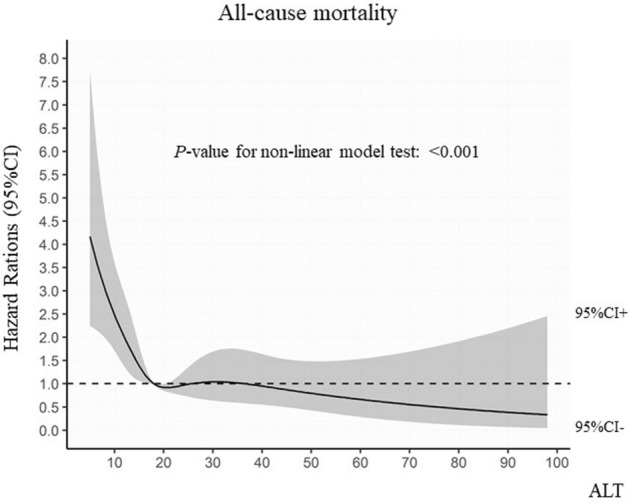
Restricted cubic spline curves with logistic regression between ALT levels with all-cause mortality. Black dashed horizontal lines represent the odds ratio of 1.0. Black lines indicate the estimated hazard ratio, and the shaded ribbons represent a 95% confidence interval (CI).

We further evaluated the non-linear relationship between ALT levels and the incidence of clinical events using restricted cubic spline curves with cox proportional hazard model. Non-linear relationship between ALT levels and the incidences of all-cause mortality were found (*P* < 0.001) (Fig. 3).

### Risk discrimination performance of lower ALT

The C statistic for prediction of all-cause mortality was 0.62 (IQR, 0.58–0.66) for the CHA_2_DS_2_-VASc score (Table 3). Adding the baseline ALT level to the baseline model of the CHA_2_DS_2_-VASc score significantly increased the C statistic, net reclassification improvement, and integrated discrimination improvement for prediction of all-cause mortality (all *P* < 0.05, Table 3).

**Table 3:** Evaluation of ability of baseline alanine aminotransferase level vs CHA_2_DS_2_-VASc score to predict all-cause mortality.

Risk score	C statistic (95% CI)	*P* value	NRI (95% CI)	*P* value	IDI (95% CI)	*P* value
**All-cause mortality**						
CHA_2_DS_2_-VASc score	0.62 (0.58–0.66)	Ref.		Ref.		Ref.
CHA_2_DS_2_-VASc score + ALT	0.65 (0.61–0.68)	0.035	0.23 (0.096–0.36)	< 0.001	0.004 (0.002–0.006)	< 0.001

*ALT* alanine aminotransferase, *CHADS*_*2*_ congestive heart failure, hypertension, age ≥ 75 years, diabetes, and stroke, *CHA*_*2*_*DS*_*2*_*-VASc* congestive heart failure, hypertension, age ≥ 75 years, diabetes, stroke, vascular disease, age 65–74 years, and male, *CI* confidence interval, *NRI* net reclassification improvement, *IDI* integrated discrimination improvement.

## Discussion

This post hoc analysis of a prospective, observational multicenter study had 3 key findings. First, low ALT in patients with AF was significantly associated with older age, lower BMI, and worse nutritional indices (lower levels of cholesterol and triglycerides and lower TCB index). Second, low ALT was strongly and independently associated with an increased risk of cardiovascular and all-cause mortality in patients with AF. Third, low ALT showed incremental prognostic relevance for the prediction of all-cause mortality when added to the conventional risk score (CHA_2_DS_2_-VASc score). Accordingly, the present study suggests that low ALT reflects the severity of aging, sarcopenia, and malnutrition and may assist in risk stratification in patients with AF.

As the general population ages, diagnosing frailty, sarcopenia, and malnutrition is becoming increasingly important in patients with common chronic diseases. Recently, the clinical significance of physical activity level and nutritional status in patients with AF has been attracting the interest of researchers^25,26^. Several recent studies showed that a low level of physical activity and malnutritional status are associated with adverse clinical outcomes^25,26^. Thus, a reliable and quick screening marker of frailty, sarcopenia, and malnutrition is needed.

Generally, ALT is of most clinical interest when the level is elevated because higher ALT levels are seen in metabolic diseases such as obesity, hyperlipidemia, and type 2 diabetes mellitus^14^. In contrast, recent clinical research revealed that extremely low ALT is associated with aging, sarcopenia, frailty, and shortened survival in the older population and in people with cardiovascular diseases^13,17^. Low ALT may be a biomarker of frailty and sarcopenia^15^. ALT is found not only in the liver but also in muscles and adipose tissue. In patients with obesity, ALT may leak into the blood from adipose tissue^27^. On the other hand, little ALT is released into the blood in patients with sarcopenia, who have low muscle mass or body fat. The present study revealed that low ALT is associated with older age and a BMI < 18.5 kg/m^2^, which is one of the diagnostic criteria of sarcopenia in the Japanese population^28^. Therefore, low ALT may reflect sarcopenia and frailty in patients with AF. In addition, low ALT is considered to reflect malnutrition status^29^. One possible explanation for this mechanism is that malnutrition often causes pyridoxine deficiency, which leads to lower ALT levels because pyridoxl-5′-phosphate is a coenzyme for transaminases^17^. In the present study, low ALT was significantly related to lower levels of cholesterol and triglycerides and a lower TCB index, a novel nutritional marker in cardiovascular diseases^21^.

The present study demonstrated that low ALT is strongly associated with high mortality risk. Previous studies have reported that AF is closely associated with an increased risk of cardiovascular and all-cause mortality compared to the general population^6,7^. Clinical factors such as older age, heart failure, and renal dysfunction are generally thought to be common prognostic factors that are related to the shorter survival rates of patients with AF, but the association between ALT values and clinical outcomes of AF has not been studied^30^. The study also showed that low ALT is significantly associated with high mortality risk after adjusting for these common prognostic factors. One possible explanation of this association is that low ALT serves as a biomarker for aging, sarcopenia, and malnutrition in patients with AF.

Adding the ALT level to the baseline model of the CHA2DS2-VASc score showed an incrementally better prognostic value. The CHA_2_DS_2_-VASc score is a well-established conventional marker of stroke risk in patients with AF. Moreover, it was recently used as a reliable risk score for all-cause mortality risk beyond thromboembolic risk^24^. The CHA2DS2-VASc score includes the effects of various clinically relevant factors, such as age, heart failure, diabetes, and complications of vascular disease, but it does not include factors related to sarcopenia or nutritional status. Thus, adding the ALT level to the CHA_2_DS_2_-VASc score provides additional prognostic information for predicting mortality in AF. In addition, it is clinically useful to use the serum ALT level as a screening tool because measurement of ALT is widely available, reliable, inexpensive, and repeatable.

Physical training and nutritional therapy may be effective treatment for patients with AF and sarcopenia and malnutrition. Recently, some studies demonstrated that higher levels of physical activity and better cardiorespiratory fitness decrease the risk of mortality in patients with AF^8^. For example, one large prospective cohort study that included 1117 patients with AF reported that physical activity and cardiorespiratory fitness were inversely associated with long-term all-cause mortality risk in patients with confirmed AF^8^. Low ALT may serve as a quick and reliable surrogate marker for early identification of patients with AF who have sarcopenia and malnutrition and, consequently, a high risk of mortality and therefore need early intervention, including physical training and nutritional therapy.

The present study has several limitations. First, although the registry had a multicenter-prospective design, the geographical area was limited mainly to Tokyo. Second, ALT levels are affected by various factors, including chronic hepatitis, alcohol consumption, and medications, such as levodopa, which is used for Parkinson disease. Furthermore, different intake levels of vitamin B6 and exercise levels might interfere with the ALT level. We could not completely exclude these factors. Third, the ALT level was assessed only at baseline; hence, changes of ALT levels and their association with clinical outcomes could not be evaluated. Last, several patients lacked baseline ALT data and were excluded from the study population, so the study may have had some selection bias.

## Conclusion

Our results suggest that low ALT may reflect aging, sarcopenia, and malnutrition and be independently associated with a high risk of all-cause mortality in patients with AF. Low ALT may be a useful screening tool for identifying poor clinical outcomes in patients with AF.

## Data Availability

The datasets generated during and/or analysed during the current study are available from the corresponding author on reasonable request.
